# Tissue toxicity following the vaginal administration of nanosilver particles in rabbits

**DOI:** 10.1093/rb/rbv016

**Published:** 2015-11-04

**Authors:** Dandan Chen, Zhaopeng Yang

**Affiliations:** Institute for Medical Devices Control, National Institutes for Food and Drug Control, Beijing 100050, China

**Keywords:** nanosilver, transmission electron microscopy, migration route

## Abstract

Nanosilver particles are used in various clinical settings because of their antibacterial properties. However, their safety evaluation when used for gynaecological disorders has not been established. Nanosilver particles were administrated in the vagina of New Zealand rabbits, and the pathological appearance of the surrounding tissue was examined by hematoxylin–eosin staining and transmission electron microscopy (TEM) after 1 and 3 days of treatment. The nanosilver content was assessed by plasma mass spectrometry, and the presence of particles in the hepatic portal vein blood was assessed by TEM. The results of our study show that the vaginal administration of nanosilver particles caused ultrastructural changes to the vaginal mucosa, urethra and rectum, with accumulation of particles in all tissues. These results demonstrate a new migration route of nanosilver particles following vaginal administration. They also demonstrate, for the first time, that the vaginal administration of nanosilver particles can enter the blood circulation system by examining the hepatic portal vein blood under the TEM which is the most direct visualized evidence.

## Introduction

Among the numerous medical nanomaterials, nanosilver has been used extensively because of its unique antibacterial properties. Despite the fact that the safety evaluation of nanosilver particles has not been clearly established, nanosilver products are currently used in various clinical applications in China.

Over the past decade, a large number of *in vivo* studies conducted in animal models have demonstrated that nanosilver particles accumulated in various organs when administered through the oral, intraperitoneal, intravenous and subcutaneous routes [1]. In mice, e.g. the intraperitoneal and intravenous administration of nanosilver particles was associated with the accumulation of particles in organs through the placental and blood-testis barriers [2]. In a subacute murine inhalation model, Stebounova *et al.* [3] showed that nanosilver particles only induced minimal lung toxicity or inflammation. Bidgoli *et al.* [4] showed that burns healed more rapidly with nanosilver wound dressing, but a rise in plasma transaminase, and mononuclear lymphoid and polymorphonuclear leukocyte infiltrations was observed around the hepatic vein. Collectively, these divergent results demonstrate that further studies are needed to determine the potential toxicity of nanosilver medications.

Different nanosilver products have been used for the treatment of gynaecological cervicitis or cervical erosion because of their antibacterial properties. However, the safety of nanosilver products following their vaginal administration has not been evaluated. In the present study, we assessed the ultrastructural morphology of the surrounding vaginal tissues. Our results highlight a new migration route of nanosilver particles, and provide a scientific basis for the risk assessment of nanosilver products in women.

## Materials and methods

### Characterization of nanosilver particles

The nanosilver particles were purchased from Alfa Aesar China (Tianjin) Co., Ltd. (Batch No. B18Z014). As assessed by transmission electron microscopy (TEM), the nanosilver particles were round in shape, and in ∼90%, the diameter ranged between 20 and 40 nm.

### Experimental model

The absorption and accumulation of nanosilver particles in surrounding tissue following vaginal administration was assessed in healthy female New Zealand rabbits weighting 2.5–3.0 kg. Based on the published literature, the typical clinical dose and practical circumstances, a dose of 0.1 g/kg of nanosilver particle was administered topically in the vagina of the rabbits.

The animals were divided into a control group and two treatment groups. The sampling time points were: (i) pre-administration (control group), (ii) 1 day (24 h) following treatment and (iii) 3 days (72 hours) following treatment. At each time point, the animals were killed, and tissue samples were taken from the vaginal mucosa near the end of the urethra, the vaginal mucosa near the end of the rectum, the urethra and the rectum. All samples were washed with normal saline, and 1/3 of each tissue samples were immersed in a 10% formalin fixation solution for hematoxylin–eosin (HE) staining, 1/3 were immersed in a 2.5% paraformaldehyde and 2.5% glutaraldehyde fixation solution for TEM sample preparation, and 1/3 were used for the detection of nanosilver particles following digestion. At each sampling time point, blood samples from the hepatic portal vein were drawn for TEM analysis.

### HE staining

All tissue samples were prepared according to standard HE staining techniques, after which their appearance was evaluated for pathological changes using an optical microscope.

### Detection of silver content

All samples were placed in a digestion tube filled with 3 ml of nitric acid, and left to be digested for 24 h. The samples were then placed in a fume cupboard and heated at low temperature until the solution liquid turned clear. The samples were heated until almost dry and left to cool. The residue in the digestion tube was dissolved in ultra-pure deionized water, and diluted to 25 ml. The amount of silver in each solution (i.e. the silver content in each sample) was analysed using plasma mass spectroscopy. The specific particle mass per unit (mg) within each different type of tissue was calculated [5, 6].

### TEM

Each sample was fixed in a 2.5% paraformaldehyde and 2.5% glutaraldehyde solution at 4°C for 2 h. The samples were then washed twice in PBS for 10 min before being placed in a 1% osmic acid fixation solution for 2 h at 4°C. Afterwards, the samples were washed three times in ultra-pure deionized water for 10 min, and dehydrated with graded ethanol. The samples were then subsequently placed in an epoxy propane–resin solution (1:1) for 1 h at room temperature, an epoxy propane–resin (1:4) for 1 h at room temperature, and a pure resin infusion for 2 h at room temperature. The samples were embedded in an Epon812 epoxy resin and cut into 1 µm semithin sections. Each section was stained with methylene blue-azure and analysed under an optical microscope. Ultra-thin sections were stained with uranyl acetate-lead citrate and analysed against a copper grid [7, 8].

### Blood sampling

About 3.0 ml of blood were drawn from the hepatic portal vein, centrifuged for 5 min at 3000 rpm, and prepared using the same method as for the TEM tissue samples.

## Results

### Localized morphological changes by HE staining

Under a standard optical microscope, the epithelial cells in the innermost part of the vaginal columnar epithelium of the mucosa were orderly and complete. Below the mucosa, cells were loosely connected to tissue, with the presence of small blood vessels surrounded by minor oedema. Surrounding the mucosa was overlapping ring-shaped smooth muscles, which contained blood vessels. The outer membrane did not feature any conspicuous inflammation.

The cross-sections of the urethra of the control animals revealed the following characteristics: some of the epithelial cells in the stratified transitional mucosa epithelium were loose and detached. Below the mucosa, cells were loosely connected to tissue, which contained blood vessels and fibrocytes. The surrounding tissue presented slight oedema, but was not conspicuously inflamed. Outer to the mucosa were intertwined smooth muscles, which also contained blood vessels. The outer membrane of the mucosa did not present any conspicuous changes.

**Figure 1.**
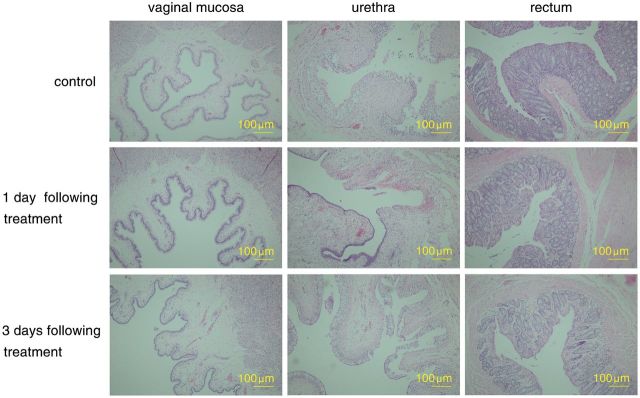
The HE staining images of the tissue of the vaginal mucosa, urethra and rectum at different time points following treatment (1 and 3 days), compared with normal tissues

The cross-sections of the rectum of the control animals revealed the following characteristics: between the epithelium and the lamina propria were multiple intestinal glands. The lamina propria contained blood vessels, as well as a few lymph nodes that were scattered around the blood vessels and which invaded cells. Outside the lamina propria were smooth muscles arranged in circular rings on the inner layer and longitudinally in the outer layer. Outside these muscles was the placenta percreta and fatty connective tissue, with no conspicuous changes (Fig. 1).

### Inductively coupled plasma—mass spectrometer (ICP-MS) detection

At 1 and 3 days following treatment, the nanosilver content in the vaginal mucosa, urethra and rectum had all increased as compared with the control group. Because the nanosilver content remained stable 3 days after treatment, it suggests that it caused a localized accumulation in surrounding tissue (Table 1).

**Table 1.** The detection of silver content using ICP-MS

Group	Vaginal mucosa	Urethra	Rectum
Control (µg/kg)	<5.0	<5.0	<5.0
1 day following treatment (µg/kg)	1537.5	601.5	<5.0
3 days following treatment (µg/kg)	1532.5	541.9	6.9

### TEM

**Figure 2.**
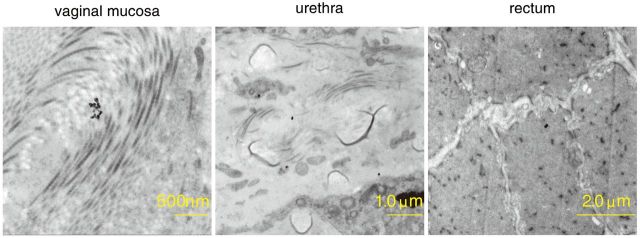
Transmission electron microscopy images of several high density round nanosilver particles in the tissue of the vaginal mucosa, urethra and rectum 1 day following treatment

**Figure 3.**
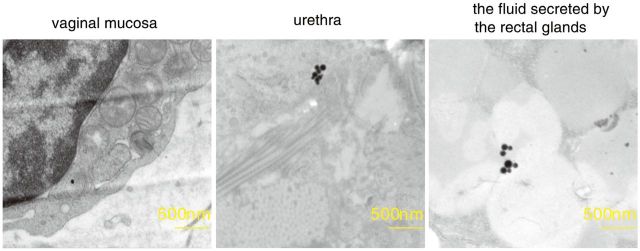
Transmission electron microscopy images of several high density round nanosilver particles in the endoplasmic reticulum of the vaginal mucosa, the tissue of the urethra and the fluid secreted by the rectal glands at 3 days following treatment

An assessment of the ultrastructural changes of the tissue surrounding the vagina was performed at 1 and 3 days following treatment with nanosilver particles. At 1 day post-treatment, several high density round nanosilver particles were observed in the tissue of the vagina mucosa, the urethra and the cells of the rectum (Fig. 2). At 3 days post-treatment, the TEM analysis revealed that the high density round nanosilver particles were swollen, and expanded in the endoplasmic reticulum of the vaginal mucosa. In the tissue of the urethra and the fluid secreted 7by the rectal glands (Fig. 3), multiple high density nanosilver particles were observed.

These results demonstrate that ultrastructural changes occur in the vaginal mucosa, the urethra and the rectum during the first 3 days following the administration of nanosilver particles. In addition, high density round nanosilver particles (ranging from a few to several) were observed in the tissue of the vaginal mucosa, the urethra and the rectum.

### TEM analysis of portal vein blood

**Figure 4.**
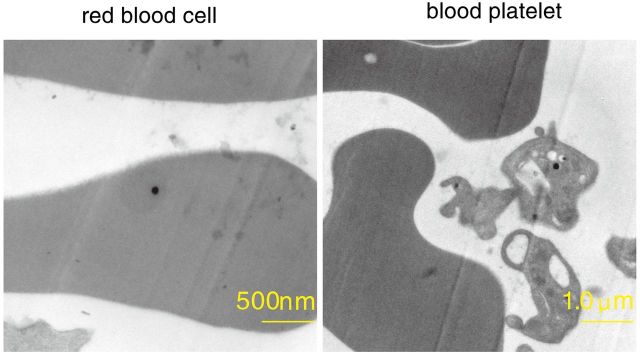
Transmission electron microscopy images of nanosilver particles forming conjugates with red blood cell and blood platelet

As with tissues, analysis at 1 and 3 days after treatment revealed several high density round nanosilver particles in the hepatic portal vein blood. Some nanosilver particles formed conjugates with red blood cell, while others formed conjugates with blood platelet (Fig. 4).

## Discussion

Most studies have evaluated the biological safety of nanosilver particles through the detection of blood silver content [9, 10] following inhalation [4, 11–13] or oral [14] and subcutaneous [9, 10] administration. Various types of nanosilver particles are currently available, and they are widely used as an antimicrobial agent for the treatment of cervicitis and cervical erosion [15–22]. However, research into the accumulation and toxicity of nanosilver particles in the tissue surrounding the vagina is lacking.

The present study revealed that, following the vaginal administration of nanosilver particles, silver accumulates in the tissue surrounding the vagina which caused ultrastructural pathological changes not only to the vaginal mucosa but also to the urethra and rectum. We also observed the migration of individual or multiple high density nanosilver particles between these tissues, thereby creating a cycle of cytotoxic reactions. Collectively, our results demonstrate new migration routes of nanosilver particles when administered intravaginally.

Transmission electron microscopy observations performed at 1 and 3 days following treatment revealed several high density round nanosilver particles in the hepatic portal vein blood, with some nanosilver particles forming bonds with red blood cell and blood platelet. Because the blood from the esophagus, stomach, intestinum tenue, intestinum crassum, pancreas, cholecyst and spleen flows through the hepatic portal vein, it is possible nanosilver particles might accumulate in more distant tissues. We are not aware of other studies demonstrating the formation of nanosilver particle conjugates with circulating blood cells. This is clearly of scientific relevance as our observations were made using TEM, the most direct visualization technique to date. By entering the blood circulation, our results suggest new migration routes of nanosilver particles following vaginal administration, and should prompt further research into their systemic distribution.

In summary, our results show, for the first time, that the vaginal administration of nanosilver particles leads to (i) their accumulation in the tissue surrounding the vagina, causing ultrastructural pathological changes, (ii) the potential promotion of cytotoxic reactions and (iii) by entering the blood circulation, it suggest additional migration routes to be elucidated.
